# Animal models of transient ischemic attack: a review

**DOI:** 10.1007/s13760-020-01295-5

**Published:** 2020-02-11

**Authors:** Jiahui Wang, Ping Zhang, Zhouping Tang

**Affiliations:** Department of Neurology, Tongji Hospital, Tongji Medical College, Huazhong University of Science and Technology, No. 1095 Jiefang Road, Wuhan, 430030 China

**Keywords:** Transient ischemic attack, Animal model, Cerebral blood flow monitoring

## Abstract

Transient ischemic attack (TIA) is defined as a brief episode of neurological dysfunction caused by focal cerebral ischemia. TIA is a critical early warning signal of stroke. Patients with TIA may have long-term cognitive decline. The pathogenesis and pathological changes of TIA have not been fully elucidated. Animal models can simulate the process of human diseases and are essential tools to investigate injury mechanisms and therapeutic approaches of TIA. Most TIA animal models are based on ischemic stroke models and the definition of TIA. Each model has unique strengths and weaknesses. The establishment of a successful and reliable TIA model should follow three criteria: (1) objective evidence of cerebral arteries occlusion and reperfusion, (2) no permanent neurological deficit, and (3) no acute cerebral infarction. However, experimental animal models are impossible to be completely consistent with human TIA, because TIA itself is a heterogeneous disease. In the present review, the selection of animals, methodological development, and evaluation of cerebral blood flow of animal models of TIA are comprehensively evaluated.

## Introduction

Transient ischemic attack (TIA) is a brief episode of neurological dysfunction caused by focal brain, spinal cord or retina ischemia, without acute cerebral infarction [1]. Because of the absence of residual neurological deficits and imaging-based evidence of cerebral infarction, TIA was previously considered a benign cerebral ischemic event. Nevertheless, recently, TIA has been found to be an important early warning signal of stroke. Up to 23% of patients with ischemic stroke have experienced TIA during the hours to days preceding the stroke [2]. Additionally, patients with TIA may exhibit long-term cognitive decline, which is a key risk factor for dementia [3, 4]. Therefore, the prevention and treatment of TIA should not be ignored. However, the actual diagnosis and treatment of TIA is not optimal. Studies indicate that the age-standardized incidence of TIA in Europe ranges from 28 to 59/100,000/year [5]. Another study reports that the age-standardized morbidity rate of TIA in China is 2.27%, but the diagnostic rate and standardized treatment rate are only 16.0% and 4.07%, respectively [6]. To improve the diagnosis and treatment strategy of TIA, it is necessary to develop an appropriate animal model that can simulate the characteristics of human TIA to enable the investigation of injury mechanisms, pathological changes and interventions for TIA. This review summarizes the selection of animals, methodological development and assessment of cerebral blood flow in animal models of TIA.

## Experimental TIA models

### Selection of species

TIA models may involve both small and large animals (e.g., mice, rats, guinea pigs, rabbits, pigs, monkeys, and baboons), and now most of the TIA modeling are performed in small animals such as mice and rats. There are some advantages of utilizing large animals to study TIA. The brains of large animals, especially non-human primates such as monkeys and baboons, are similar to the human brain in structure and function, which may enable the experimental results obtained from large animals to more effectively translate to clinical applications. However, large animal models are costly and involve ethical restrictions. It is also difficult to establish a stable TIA model in large animals due to collateral circulation and abundant vascular anastomosis [7].

Compared to large animals, the use of small animals presents distinct advantages, including an accessible supply, lower cost, less ethical restrictions, and most of all, the ease of replicability. The rat is one of the most commonly used animals in TIA studies due to the following advantages: (1) its similarities to humans in cerebrovascular structure [8], (2) the ability to use neurological and behavioral measurements to evaluate the severity of cerebral ischemic injury [9], (3) its moderate body size which facilitates the monitoring of physiologic parameters, and (4) its small brain size which allows for in vivo freezing techniques for metabolite studies [10]. In addition, since the mouse has an established genetic foundation and can be genetically homogeneous, it is widely used in transgenic technology studies to further explore the molecular pathology of TIA [11, 12]. However, there are significant differences between small animals and humans in brain structure and function (e.g., size, gray matter distribution) [13], therefore, the pathological characteristics of small animal TIA models may differ greatly from human TIA.

In addition to animal species, the gender and age of animals can also affect the experimental results of TIA models. Since the estrogen has a neuroprotective effect which can alleviate cerebral ischemic injury [14, 15], male animals are used in most studies. Older animals exhibit pathological changes in the carotid artery, such as vascular tortuosity, hardening and lumen stenosis [16], which may confound a TIA model. Thus, older animals are typically not used.

Male adult rats are the most commonly used animals for the preparation of TIA models, but there is currently no unified standard for species selection. Due to the diversity of anatomical structures, functions and physiological characteristics of different species, established models differ in pathological mechanisms and disease manifestations. In practical applications, one should comprehensively consider the design, purpose, and funding of a study and try to choose economical and available animals with simple structures and similar characteristics to human diseases to achieve reliable results.

### Model selection

TIA animal models are rare in the literature, most of which are based on ischemic stroke models and the definition of TIA. There is currently a lack of models specifically for establishing TIA. In this section, we would like to describe all the ischemic models available and present the possibility for their use in TIA modelling. Among the ischemic models, the middle cerebral artery occlusion (MCAO) is the most widely used method for inducing TIA, including craniotomy and intraluminal suture models. There are also photothrombosis, endothelin-1, embolic and platelet aggregation models (Table 1).

**Table 1:** Common animal models of ischemia and their use for TIA modeling

Model	Method	Features	Applications	Use for TIA modeling	Details of inducing TIA
Craniotomy model	Mechanically interrupts the distal blood flow of the MCA	Good reproducibility Visual confirmation of successful MCAO	Histopathological study of TIA	Yes	Occludes the distal MCA with microvascular clip for 15 min [21]
Intraluminal suture MCAO model	Blocks the origin of the MCA by a suture	Highly controllable ischemia-reperfusion	Histopathological study of TIA	Yes	Different blocking duration (Table 2)
Photothrombosis model	Irradiates the exposed skull after the injection of a photoactive dye	Desired brain ischemic lesions	Study of antiplatelet, anticoagulation and endothelium protection therapy	Yes	Injects Rose Bengal (10 mg/ml in concentration) into the tail vein at 0.2 ml/100 g body weight and illuminates for 3 min using a 5 mW, 532 nm CW laser light [27]
Endothelin-1 model	Injects ET-1 into the brain parenchyma	Purely ischemic injury Localized and specific lesions	Histopathological study of cerebral ischemia	No	/
Embolic model	Injects fresh clots or lipid microparticles into the carotid artery	Mimics human TIA more closely	Study on the treatment of TIA	Yes	Injects 0.5 mg of melted solid lipid microparticles (75–90 μm in diameter) via a carotid artery catheter [32]
Platelet aggregatio*n* model	Infuses collagen fibrils or ADP into the carotid artery	More variability in the experimental outcomes	Study on the mechanism of TIA	Yes	Infuses ADP (8 mg per min for 5 min) into the carotid artery [33]

*TIA* transient ischemic attack, *MCA* middle cerebral artery, *MCAO* middle cerebral artery occlusion, *ET-1* endothelin-1, *ADP* adenosine diphosphate

### MCAO model

The middle cerebral artery (MCA) is the most frequently affected cerebral vessel in human ischemic cerebral vascular disease. Therefore, models that occlude this artery have the greatest translational potential.

### Proximal MCAO model

The proximal MCAO model is also known as the intraluminal suture MCAO model, which is currently the most common method for TIA modeling. This method involves inserting a suture into the internal carotid artery (ICA) and advancing it until it blocks the origin of the MCA, causing a sharp decrease in the blood flow to the frontoparietal cortex and striatum. According to the path of the suture into the ICA, this model can be divided into two categories: the common carotid artery (CCA) approach and the external carotid artery (ECA) approach. The CCA approach is relatively simple to perform, but the reperfusion is achieved by the blood supply from contralateral cerebral circulation via the circle of Willis, which alters cerebral hemodynamics [17]. Therefore, the ECA approach is more consistent with clinical practice because it retains the anatomical integrity required for reperfusion.

The intraluminal suture MCAO model requires no craniotomy and is minimally invasive. Its greatest advantage is the ability to precisely control the duration of ischemia and efficiently initiate reperfusion by withdrawing the suture. However, the suture model has several disadvantages. First, it is difficult to confirm the success of this model without the help of cerebral blood flow (CBF) monitors. Second, the suture may interrupt the other branches of the ICA while occluding the MCA, resulting in additional ischemic injury. Third, the carotid intima may be damaged by the suture [18], which can cause thrombosis and affect histopathological results. Fourth, the cerebral vessels suddenly and completely recover recanalization after the suture is withdrawn, which is inconsistent with the hemodynamic characteristics of TIA patients. Additionally, this model shows no response to anticoagulant and antiplatelet therapy [19], and thus it is not an ideal model for studying the prevention and treatment measures of TIA. Finally, the reproducibility of this model is influenced by many factors, such as suture diameter and coating. The insertion length of suture also affects the degree of CBF decline, causing different ischemic injury. However, in recent years, with the application of CBF monitors in the modeling process, the consistency and success rate of this model have been greatly enhanced. CBF monitors not only help to avoid insufficient ischemia but also reduce the occurrence of complications (e.g., subarachnoid hemorrhage).

It is worth noting that the infarct thresholds (the minimum duration of ischemia that can cause cerebral infarction) vary in different animals used in the suture method of TIA. The mechanism for this variation is still unclear and may be caused by many factors, including differences in the composition of the circle of Willis, the distribution of gray and white matter, neuron density and other neuromorphological features [20]. Furthermore, because of individual differences in the formation of collateral circulation, the same strain may present different infarct thresholds. Additionally, the experimental results of the suture method vary greatly, and the filament, anesthesia and even the operation skill will affect the reproducibility of this model. In conclusion, the reasons for the different infarct thresholds among species need to be further explored. When this method is used in a TIA model, a pilot experiment should be conducted to clarify the infarct threshold of this animal (Table 2).

**Table 2:** Infarct thresholds of several mouse and rat strains (suture method)

Species, strain	rCBF during occlusion (%)	Infarct threshold	The duration of MCAO for TIA model (min)
Rat, SD [18]	20	10 min Hippocampal infarcts were observed in 33.3% (2/6) of rats*	< 5
Rat, Wistar [20]	24	12.5 min Caudate nucleus infarcts were observed in 16.7% (1/6) of rats*	< 10
Mouse, NMRI [35]	15	12.5 min Cortical infarcts were observed in 16.7% (1/6) of mice*	< 10
Mouse, ICR-CD1 [36]	15	10 min Striatal infarcts were observed in 50.0% of mice*	< 10
Mouse, C57BL/6 [37]	34	No infarcts were found in any mice after MCAO for 5 min*	

*rCBF* relative cerebral blood flow, which is the percentage of CBF in the basal CBF

*Infarcts that were confirmed by MRI; as shown in the table, 10 min can be selected for TIA-related study in the pre-experiment

### Distal MCAO model

The distal MCAO model is also referred as the craniotomy model. In this model, the MCA is exposed firstly via the temporal or orbital route. The distal blood flow of the MCA is then temporarily interrupted by microvascular clip [21], balloon or blunt micropipette compression [22, 23], microsurgical hook lift [24] or suture ligation [25], causing the cortical ischemic injury.

The greatest advantage of using this method to establish a TIA model is that the different mouse and rat strains currently studied (including spontaneously hypertensive rats [21], Swiss albino mice [22, 23] and Wistar rats [24]) have consistent infarct thresholds. Specifically, no cerebral infarction confirmed by MRI occurs when the MCA is occluded for less than 15 min. Thus, when establishing a TIA model with this method, one can choose “15 min” for a preliminary experiment to further explore the exact infarct threshold. Additionally, in this model, the MCA is blocked under direct microscope observation, and so it has high reliability, high success rate and good repeatability.

The disadvantages of this model mainly relate to invasive craniotomy, which involves the risk of intracranial infection, and may cause MCA or cortical tissue damage. Furthermore, the procedure requires skilled microsurgery techniques. These disadvantages limit the broad application of this method in TIA modeling to some extent.

### Modified photothrombosis model

This model is based on the principle of intravascular photo-oxidation. A light-sensitive dye (e.g., Rose Bengal) is injected intravenously or intraperitoneally, and after irradiating the exposed skull with the light of a specific wavelength, oxygen radicals are generated, which induce vascular endothelium damage, platelet aggregation, and thrombosis [26], thereby resulting in cortical ischemic injury. This method is usually used to model ischemic stroke. However, Liu et al. [27] optimized the relevant parameters and developed a TIA rat model. They decreased the light intensity (5 mW) and reduced the illumination duration (3 min), which caused unstable microthrombus formation in selected cortical vessels, with CBF dropping to 24% of the baseline level. Within 1 to 2 h post-ischemia, the thrombus began to dissolve spontaneously. At 4 h post-ischemia, reperfusion was observed, and the CBF returned to 88% of the baseline level, accompanied by the restoration of neurovascular function. The whole process was similar to clinical TIA. Unfortunately, TTC staining at 24 h showed that there were still tiny infarcts in the cortex, which might be associated with silent lacunar infarcts, so this model differs from the clinical definition of TIA, characterized by the absence of infarction.

In contrast to the MCAO model, the photothrombosis model can produce ischemic injury in specific brain regions. In addition, this model is minimally invasive and the survival rate is high. The size and depth of ischemic injury can be controlled by adjusting the dye concentration, light intensity and illumination duration. Furthermore, the process resembles human cerebral thrombosis, so this model can be used in the study of antiplatelet, anticoagulation and endothelium protection therapy. However, the photothrombosis model requires complex experimental equipment, and direct damage to brain tissue is a substantial risk, which may influence histopathology results. Another limitation of this model is that while the infarct volume of the modified method is significantly reduced compared to the traditional method, due to thrombosis and the inability to achieve controllable reperfusion, the occurrence of cerebral infarction cannot be avoided, which is inconsistent with the clinical definition of TIA. Therefore, this is not a recommended model of TIA.

### Endothelin-1 model

Endothelin-1 (ET-1) is a potent and reversible vasoconstrictor peptide which can be used to induce cerebral ischemia models. Following stereotaxic intracerebral injection of ET-1, CBF is significantly decreased (50%), then recovers to baseline levels over several hours [28]. Horie et al. [29] injected the same dose of ET-1 into the brain parenchyma of SD rats and four different mouse strains, inducing large and reproducible lesions in rats, with no lesions in mice. The reason for this difference may be the different expression of ET-1 receptor isoforms in mice and rats, resulting in different responses of rats and mice to ET-1. As we know, the mouse brain is rich in endothelin-B receptor which mediates vasodilation, leading to the poor ET-1-mediated cerebral vasoconstriction and an inability to induce cerebral infarction. This is consistent with the pathological characteristics of TIA. However, due to the absence of CBF monitoring in this study, it is not clear whether effective CBF decline occurred after ET-1 injection in the mice. Therefore, further investigation to determine whether the mouse ET-1 model can be used for TIA-related research is warranted, but at present, this model has only been applied to rats.

Compared with the MCAO model, the ET-1 model can induce focal ischemia in superficial or deep brain regions, with lesions that are more localized and specific. This model can also be optimized by adjusting the corresponding parameters, such as the dose and infusion mode of ET-1 to control the severity and duration of ischemia. Importantly, the pathological changes of this model are specifically caused by ischemic injury rather than inflammatory reactions [28], which may be more relevant to clinical TIA pathology. However, ET-1 generally causes severe cerebral ischemia (except in mice) due to its robust vasoconstrictive effects, and induces cerebral infarction. Therefore, strictly speaking, this model is not a specific TIA model.

### Embolic model

The embolic model can be divided into two categories: thromboembolic and non-clot embolic models. In the thromboembolic model, a fresh clot is used for the establishment of a TIA model, as older clot is resistant to thrombolysis and cannot autolysis, which can cause extensive cerebral infarction. In the non-clot embolic model, a lipid microparticle is the most common embolus.

### Fresh clot model

Microembolism is considered a major cause of TIA, and thus the thromboembolic model has strong face value. Culp et al. [30] directly injected fresh clots (1.0 mm length, 0.6 mm diameter) into the ICA of rabbits. Angiography revealed that the injected clots flowed to the MCA in most rabbits, but 46% of cases presented incomplete MCA occlusion or no visible occlusion. TTC staining found that 60% of cases had no infarcts, possibly resulting from prompt autolysis of the clots and vascular recanalization, or adequate vasodilation or collateral development. This model mimics human TIA more closely than other models and is a valuable tool for studying TIA-related treatment.

Disadvantages of the thromboembolic model include: (1) the distribution of clots in cerebral vessels is indefinite, and the volume and location of lesions vary, (2) reperfusion is uncontrollable and the duration of ischemia cannot be reliably determined, (3) the rate of intracerebral hemorrhage and mortality are high.

### Lipid microparticle model

The physicochemical properties of lipid microparticles are similar to those of lipid-rich emboli derived from atherosclerotic plaques, which are the major source of microembolism, and thus this model also simulates clinical TIA. Based on its characteristics of homogenous sphere appearance and rapid and consistent dissolvability [31], Tsai et al. [32] designed a temperature-sensitive solid lipid microparticle with a melting point approximating body temperature, which could melt rapidly after being injected into the body. After injecting these microparticles into awake rats via a carotid artery catheter, the CBF was immediately decreased and recovered completely after about 1 h. In the result, the rats were divided into neurologic symptom durations < 24 h, 24 ~ 48 h and ≥ 48 h groups, and the infarct volume was different in each group. Among these categories, the < 24 h group without cerebral infarction could be used as TIA model.

Unlike other TIA models, the lipid microparticle model can be prepared without anesthesia, which not only avoids the influence of anesthetic drugs on the experimental results but also allows for the immediate evaluation of the neurologic deficit during TIA and tracking of neurological function recovery in real time. However, this model has excessive variability and its success rate of simulating TIA is low, so the application of this model in TIA-related research is limited.

### Platelet aggregation model

The platelet aggregation model involves the infusion of certain agents (e.g., collagen fibril, ADP) into the carotid artery, which reversibly causes platelet aggregation and platelet thrombosis, eventually inducing transient cerebral ischemia. This model also presents significant variability in the duration and severity of ischemia.

### Collagen fibril model

Fritz et al. [7] infused 0.025 ml/kg collagen fibrils into the ICA circulation of baboon via an ECA cannula, producing reproducible clinical and EEG features resembling those of TIA patients. It was speculated that TIA might result from the release of chemical substances from atherosclerotic plaques which activated the prostaglandin cascade and led to intravascular platelet aggregation. This model successfully replicates the EEG features of clinical TIA and can be used to explore the mechanism of TIA. However, due to the absence of CBF monitoring, neurological assessment and imaging examination, its effectiveness as a TIA model requires additional investigation.

### ADP model

Fieschi et al. [33] successfully developed a rabbit TIA model by infusing ADP into the carotid artery. They observed platelet thrombosis in the cerebral circulation, reduced CBF, and a significant decrease in systemic blood pressure during ADP infusion. However, upon cessation of the infusion, the platelet thrombi were fragmented and blood pressure was quickly restored. Unlike with collagen fibrils, ADP can not only promote platelet aggregation but also induce systemic hypotension, causing decreased blood flow in the collateral circulation and inadequate compensation for ischemia, thus exacerbating cerebral ischemia and affecting histopathological outcomes. In addition, severe systemic hypotension may limit subsequent studies.

## Cerebral blood flow monitoring methods

Regardless of the method used to develop a TIA model, it is difficult to ensure adequate cerebral ischemia–reperfusion. CBF monitoring can not only objectively evaluate the occlusion or recanalization of cerebral arteries, but also determine the duration of ischemia, which is an essential tool for preparing and validating a TIA model [34]. Furthermore, by monitoring CBF during the establishment of a TIA model, the success of the model can be assessed in a timely manner, and the validity and reliability of the experimental results can be confirmed. Currently, the methods for monitoring CBF include laser Doppler flowmeter (LDF) monitoring, laser speckle contrast imaging (LSCI), magnetic resonance angiography (MRA), iodine-antipyrine autoradiography, and hydrogen clearance. Among them, LDF is the most widely used method. In addition, new devices such as micro-electrocorticography-functional photoacoustic microscopy system (μECoG-fPAM) and functional ultrasound (fUS) are also used. In practice, one should weigh the costs and benefits based on experimental needs, species, and modeling methods, and try to choose an appropriate, simple and convenient CBF monitoring method (Table 3).

**Table 3:** Comparison of cerebral blood flow monitoring methods in TIA models

	Theory	Advantages	Disadvantages
LDF	Laser Doppler frequency deviation	Easy operation	Limited to the cortical surface measurement*
		Low invasiveness	Single point measurement^#^
		High sensitivity	Relative measurement value
		Ability to monitor CBF quickly, continuously, and in real time	Highly susceptible to environment and activities (e.g., indoor light, surgery operation, animal breathing)
LSCI	Random interference	Non-contact	Limited to the cortical surface measurement
		Minimally invasive	Not suitable for monitoring CBF continuously
		Broad measurement range	Relative measurement value
		High spatio-temporal resolution	Requires thinning the skull for animals with thick skulls (high technical difficulty, and not conducive to long-term measurement)
		Ability to measure multiple microcirculation parameters (e.g., vascular diameter, vascular density)	
MRA	Inflow enhancement effect	Noninvasive	High requirements on equipment
		Ability to display cerebral vessels clearly	High cost
		Visual observation of cerebral vascular occlusion or patency	Inability to monitor CBF in real time
Hydrogen clearance	Tracer removal theory	Quantitative measurement	High invasiveness
		High reliability	Unable to monitor CBF continuously
		Low requirements on equipment	
μECoG-fPAM [27]	Electrophysiological function and microvascular resolution	Ability to monitor blood flow dynamics of deep cerebral vessels*	Requires complex equipment
		High spatio-temporal resolution	Requires a high level of operational skill
		Visual observation of cerebral vascular morphological changes	
fUS [38]	Pulse Doppler technology	Ability to monitor CBV of deep cerebral vessels*	Relative measurement value
		Broad measurement range^&^	Unavoidable measurement error
		High spatio-temporal resolution (100 μm and 400 ms, respectively)	Partial loss of blood flow data caused by the removal of blood vessels with slow flow rates
		Suitable for real-time monitoring of CBF indifferent brain regions	
		Requires no anesthesia	

*LDF* laser Doppler flowmeter, *CBF* cerebral blood flow, *LSCI* laser speckle contrast imaging, *MRA* magnetic resonance angiography, *μECoG-fPAM* micro-electrocorticography-functional photoacoustic microscopy system, *fUS* functional ultrasound, *CBV* cerebral blood volume

*The measurement depth of LDF, μECoG-fPAM and fUS is 1 mm, 3 mm and 8 mm, respectively

^#^the measurement range of LDF is only 1 mm^3^

^&^the measurement width of fUS up to 12.8 mm

## Conclusion

Animal models can mimic human TIA and are essential tools for studying the pathogenesis, pathology and treatment of TIA. However, the basic research on TIA is limited, and methods are still being developed. According to the definition of TIA, the establishment of a successful and reliable TIA animal model should follow three criteria [20, 35]: (1) objective evidence of cerebral arteries occlusion and reperfusion, (2) no permanent neurological deficit, (3) no acute cerebral infarction. The importance of the first criterion is to confirm the occurrence of transient cerebral ischemia by monitoring CBF and to promptly exclude the animals with inadequate occlusion or inappropriate reperfusion. The necessity of the second and third criterion relies on a neurological function test and MRI examination (both at 24 h of reperfusion), making animal models more similar to clinical TIA, and these are also the essential differences between TIA and ischemic stroke models. In summary, the various modeling methods described in this review have unique advantages and disadvantages and should be selected according to specific experimental objectives. In the future, researchers should further optimize these methods based on the above standards to develop an optimal TIA model with minimal invasiveness, good reproducibility, accessible methods, and high translational potential.
